# Controlled hierarchical self-assembly of networked coordination nanocapsules *via* the use of molecular chaperones†

**DOI:** 10.1039/d0sc05002d

**Published:** 2020-10-28

**Authors:** Xiangquan Hu, Sisi Feng, Jialei Du, Li Shao, Jinxin Lang, Chen Zhang, Steven P. Kelley, Jian Lin, Scott J. Dalgarno, David A. Atwood, Jerry L. Atwood

**Affiliations:** Department of Chemistry, University of Missouri-Columbia 601 S College Ave Columbia MO 65211 USA atwood@missouri.edu; Key Laboratory of Chemical Biology and Molecular Engineering of Ministry of Education, Institute of Molecular Science, Shanxi University Taiyuan 030006 P. R. China ssfeng@sxu.edu.cn; Institute for Advanced Interdisciplinary Research, University of Jinan Jinan 250022 P. R. China ifc_dujl@ujn.edu.cn; School of Chemistry, Xi'an Jiaotong University Xi'an 710049 P. R. China; Department of Chemical and Biomolecular Engineering, North Carolina Sate University Raleigh North Carolina 27695 USA; Department of Mechanical and Aerospace Engineering, University of Missouri-Columbia 601 S College Ave Columbia MO 65211 USA; Institute of Chemical Sciences, Heriot-Watt University Riccarton Edinburgh EH14 4AS UK; Department of Chemistry, University of Kentucky Lexington KY 40506 USA

## Abstract

Supramolecular chaperones play an important role in directing the assembly of multiple protein subunits and redox-active metal ions into precise, complex and functional quaternary structures. Here we report that hydroxyl tailed *C*-alkylpyrogallol[4]arene ligands and redox-active Mn^II^ ions, with the assistance of proline chaperone molecules, can assemble into two-dimensional (2D) and/or three-dimensional (3D) networked 
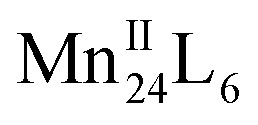
 nanocapsules. Dimensionality is controlled by coordination between the exterior of nanocapsule subunits, and endohedral functionalization within the 2D system is achieved *via* chaperone guest encapsulation. The tailoring of surface properties of nanocapsules *via* coordination chemistry is also shown as an effective method for the fine-tuning magnetic properties, and electrochemical and spectroscopic studies support that the 
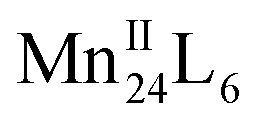
 nanocapsule is an effective homogeneous water-oxidation electrocatalyst, operating at pH 6.07 with an exceptionally low overpotential of 368 mV.

## Abstract 2

Molecular chaperones play a critical role in directing the assembly of  nanocapsules that assemble into 2D or 3D coordination networks.

## Graphical abstract

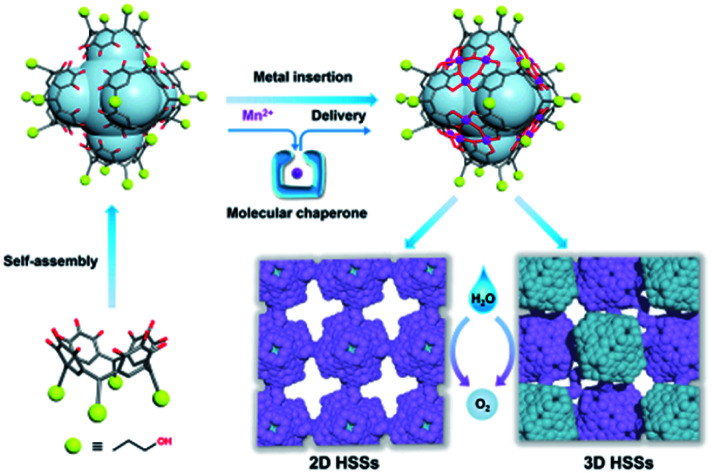

## Introduction

Hierarchical self-assembly *via* metal coordination is a ubiquitous process for constructing sophisticated supramolecular structures in nature.^1^ As an example of its use in biological systems, metal coordination or bridging plays a crucial role in folding and assembling multiple protein subunits into precise, complex and functional quaternary structures (such as viral metalloproteins).^2,3^ Metallosupramolecular assemblies such as metal–organic nanocapsules (MONCs) and/or nanocages are potentially useful models for such complex biological processes,^4,5^ and are also promising with regard to energy storage,^6–10^ molecular encapsulation,^11–15^ catalytic,^16–18^ and biomedical applications.^19,20^ To date, synthetic chemists have been able to isolate discrete cages consisting of more than 100 precisely designed units through metal coordination.^21^ A long-standing challenge, however, is the rational combination of simple components to form hierarchical superstructures with a similar level of assembly complexity as proteins.^22,23^ Another challenge that has seldom been addressed in the literature is redox-controlled metal-directed assembly. Albeit at a higher level of complexity, living organisms are able to rapidly select the oxidation state of metal ions such as Cu, Mn and Fe, with regard to protein subunit folding and assembly of quaternary structure, often with the aid of supramolecular chaperones.^24–26^ These metallochaperones are typically employed to capture, protect and insert the highly active metal ions into the specific coordination sites before elements of the quaternary structure have formed through subunit self-assembly.^26^ The powerful self-assembly approach utilised by biological systems may thus provide access to new hierarchical superstructures (HSSs) with unique properties.

Our group (and others) have used *C*-alkyl-pyrogallol[4]arenes (PgC_*n*_, where *n* is the number of carbon atoms in the pendant alkyl chains), bowl-shaped polydentate macrocycles, to synthesise MONCs *via* metal insertion.^13,27,28^ This approach gives rise to large, discrete cages which typically have one of two highly conserved structures: a dimeric cage composed of 2 PgC_*n*_s seamed/bridged by 8 metal ions, or a hexameric cuboctahedral analog comprising 6 PgC_*n*_s and 24 metal ions (the latter of which form 6 triangular faces). These MONCs are readily accessible *via* ambient or solvothermal syntheses using redox stable metal ions such as Zn^II^, Ni^II^, Ga^III^.^28–30^ Variations in structure are also possible, for instance by replacing some pyrogallol rings with resorcinol in the PgC_*n*_ framework, giving mixed macrocycles that cause ‘defects’ in the perfect MONC structure.^29^ Despite the fact that these two general supramolecular architectures accommodate metals of different size and charge, the controlled assembly of redox-active transition metals has proven difficult. For instance, it has been shown that the reaction of Fe^II^ or Mn^II^ ions with PgC_*n*_s rapidly yielded MONCs with metal ions in mixed oxidation states.^31,32^ Indeed, the assembly of mixed-valence MONCs, such as Mn^II^/Mn^III^, should be more kinetically favored than solely Mn^II^-based analogs since Mn^II^ is more thermodynamically stable and kinetically labile than Mn^III^ for coordination.^26^ We only recently achieved the assembly of Co^II^ hexameric MONCs by using a route inspired by zinc-finger proteins (ZNFs).^33^ In that case the Zn^II^ ion was used to direct assembly of hexameric MONCs that were spontaneously transmetallated with Co^II^ ions to afford the target assembly. Such results indicate that new MONCs with redox-active functionality may (as can be the case with biological systems) require additional templates or chaperones to control their assembly into the correct state.

In this context, we are encouraged to challenge the synthesis of HSSs constructed from *C*-propan-3-ol-pyrogallol[4]arene (PgC_3_OH) and coordination-inert but redox-active Mn^II^ ions; the hydroxyl group on PgC_3_OH can link MONCs to obtain HSSs.^30^ This may not only help to develop a better understanding of the redox-based self-assembly of metalloproteins, but also the construction of HSSs with emergent properties, such as magnetism and catalysis, based on the oxidation state distribution of the metal ions.^34–36^ Several reaction conditions and methodologies have been investigated to this end, yet all failed to deliver the selective assembly of any anticipated HSSs (see ESI† for details). We hypothesised that *in situ* redox reactions may prevent the formation of such highly intricate structures.

**Scheme 1:**
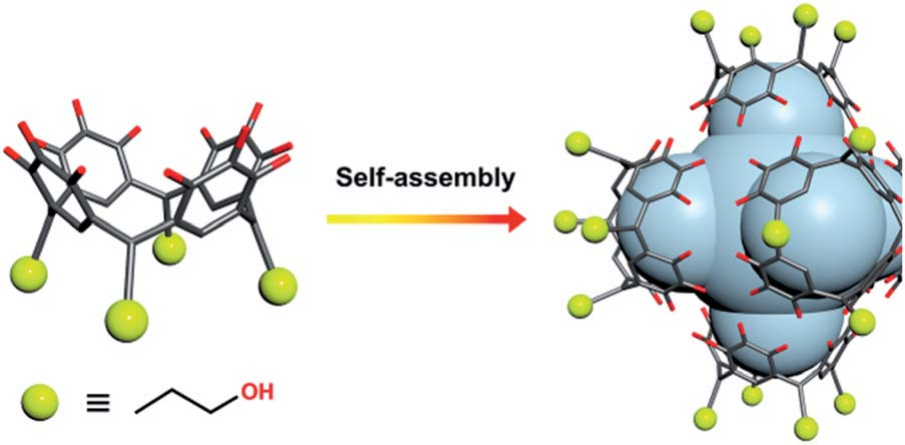
Pre-assembly strategy of Mn^II^-seamed MONC subunits used in this study. Color codes: carbon, grey; oxygen, red; Mn^II^, purple.

Herein, we present a design strategy for the construction of such otherwise unobtainable HSSs that uses a reaction system consisting of PgC_3_OH, Mn^II^ ions, and proline. The use of proline was inspired by the Mn^II^ coordination sphere in manganese-based proteins, which may effectively capture and stabilise the free metal ion, as well as modulating its weak coordination ability with regard to metal insertion.^26,37,38^ We propose a system in which PgC_3_OH is assembled into hexameric hydrogen-bonded nanocapsules (MONCs), whilst proline molecules act as the molecular chaperones to capture, protect and insert the Mn^II^ ions into the framework (Scheme 1). Once formed, the thermodynamically and kinetically very stable MONCs serve as subunits (secondary structures) and organise into more complex HSSs through the formation of intermolecular metal–hydroxyl coordination bonds. Using this approach, we obtained 2D and 3D HSSs consisting of Mn^II^-seamed MONC subunits (**1**, [Mn_24_(PgC_3_OH)_6_(H_2_O)_44_] and **2**, [Mn_24_(PgC_3_OH)_6_(H_2_O)_44_(CH_3_CN)_2_]), structurally controlled by subtle changes in reaction conditions.

## Results and discussions

**Fig. 1:**
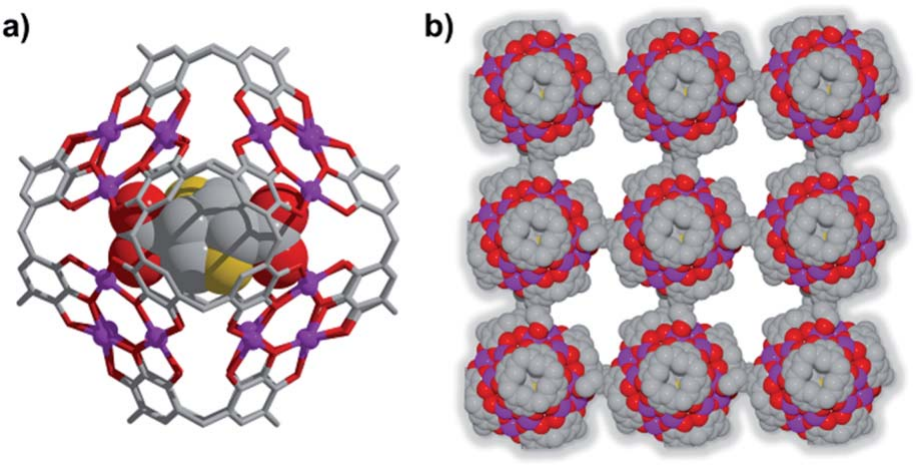
(a) Side and (b) extend views of the single crystal X-ray structure of **1**. Inspection shows 2D HSSs composed of Mn^II^-MONC subunits, each of which encapsulates two proline chaperone molecules *via* metal coordination. Color codes: manganese (purple), carbon (grey), oxygen (red), nitrogen (yellow). Hydrogen atoms, axial water ligands and hydroxyl tail alkyl chains not involved in metal–ligand coordination to adjacent MONC subunits were removed for clarity.

Compound **1** has been studied and characterised by scanning electron microscopy images (Fig. S1†), single-crystal X-ray diffraction (SC-XRD, Fig. S2†), FT-IR (Fig. S3†), elemental analysis (EA), MALDI-TOF MS (Fig. S4†), thermogravimetric analysis (TGA, Fig. S5†) and differential scanning calorimetry (DSC, Fig. S6†), details of which can be found in the ESI.† Compound **1** crystallises in the monoclinic space group *P*2_1_/*n*. The crystal structure of **1** shows a 2D framework constructed from infinite MONCs subunits, with each MONC being assembled from 30 components: six PgC_3_OH molecules and 24 metal ions (Fig. 1). The overall geometry of the MONC subunit corresponds to that of a truncated octahedron, which is similar to the previously reported hexameric MONCs.^28^ Each hexagonal face of the MONC is capped by one [Mn_3_O_3_] trimetallic cluster with Mn–O distances in the range of 2.03–2.11 Å, Mn–O–Mn angles in the range of 133.17–137.50°, and O–Mn–O angles in the range of 99.34–105.66°. All Mn^II^ ions adopt an octahedral ligand field, where the equatorial positions are coordinated with oxygen atoms from the upper-rim of PgC_3_OH units. Bond-valence sum (BVS) analysis, coupled with examination of bonding energy reveals that all Mn ions are in the +2 oxidation state (Table S1 and Fig. S7†). Interestingly, each MONC encapsulates two proline chaperone ligands that coordinate in a bridging mode between two Mn^II^ ions (metal–carboxyl distances in the range of 2.24–2.27 Å). This suggests that the proline molecules perform the critical function of a molecular chaperone, capturing, protecting and inserting Mn^II^ ions into MONCs *via* ligand exchange during the assembly process. The extended view of **1** shows that each MONC is connected to four adjacent symmetry equivalents *via* double manganese–hydroxyl coordination (M–O distances: 2.26–2.35 Å). One MONC provides a hydroxyl tail and a metal coordination site for another, and the other axial positions are occupied by water molecules.

**Fig. 2:**
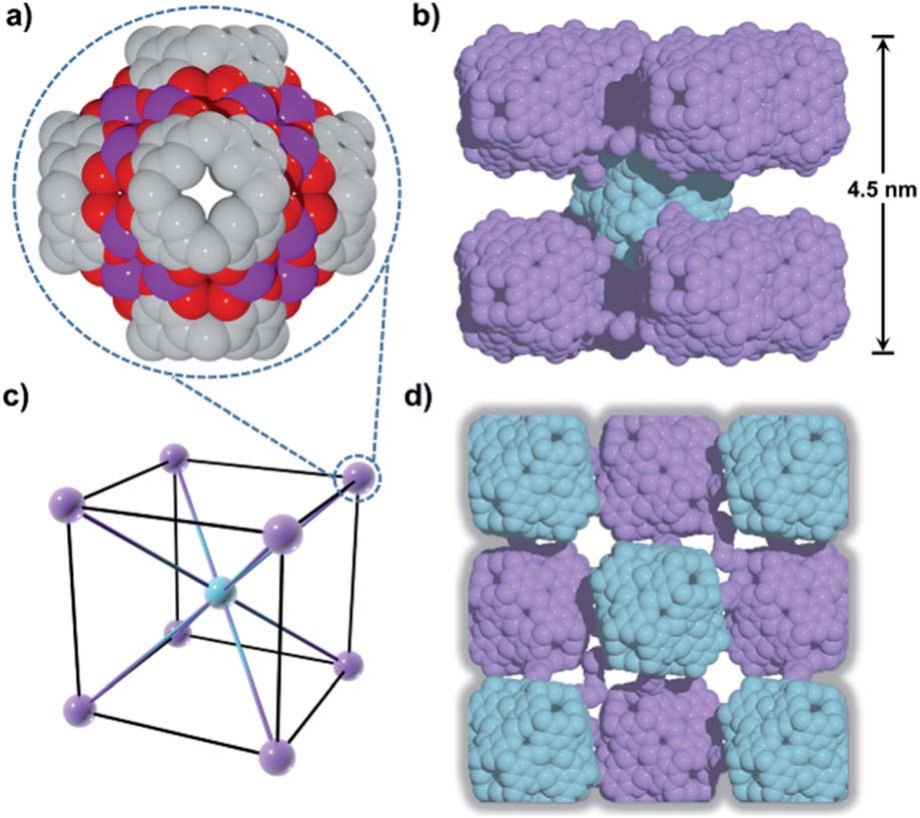
(a) Mn^II^-seamed MONC subunits (secondary structure). (b) Supramolecular nanocubes (tertiary structure). (c) CsCl unit cell and (d) the 3D hierarchical CsCl-like superstructure (quaternary structure). Hydrogen atoms, axial ligands and hydroxyl tail alkyl chains not involved in metal–ligand coordination to adjacent MONC subunits were removed for clarity.

Introduction of a greater amount of water to similar reaction conditions as those used in the synthesis of **1** changed both the internal and external properties of the Mn^II^-seamed MONCs, resulting in the formation of a 3D HSSs which crystallises in an orthorhombic system (structure solution in space group *Pccn*, **2**, Fig. 2, Table S2 and Fig. S8–S10†). On the internal surface, all axial positions at the metal centres are occupied by water molecules, whilst inspection of the exterior reveals that each MONC subunit is linked to eight symmetry equivalents *via* single manganese–hydroxyl coordination bonds (two crystallographic M–O distances: 2.276 and 2.279 Å, respectively), the result being assembly into a cubic tertiary structure (Fig. 2b). This supramolecular nanocube is assembled from 216 Mn^II^ ions and 54 PgC_3_OH macrocyclic ligands and has an edge of 4.5 nm. Within the nanocube there are two types of MONC subunits with different orientation in the solid lattice, highlighted by the disparate colours in Fig. 2. This structural motif is similar to the unit cell of CsCl (Fig. 2c), and the extended view exhibits a hierarchical CsCl-like superstructure (Fig. 2d).

**Fig. 3:**
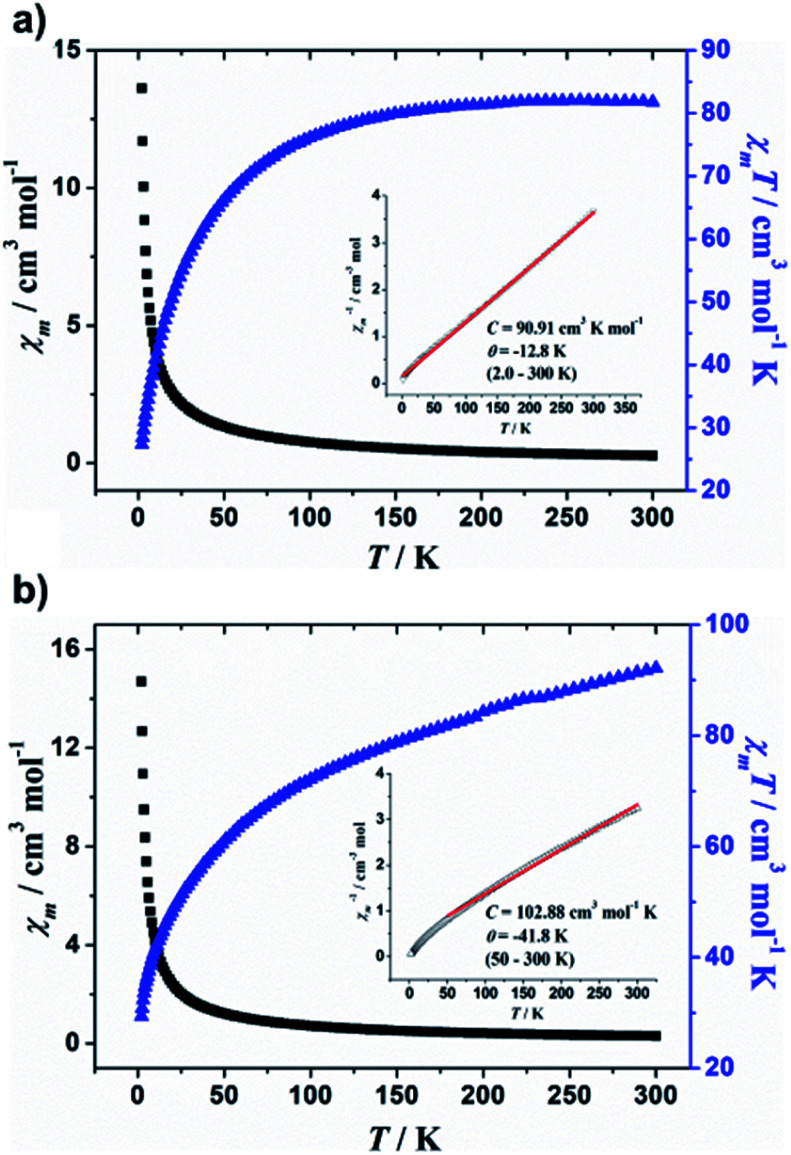
Temperature dependence of *χ*_m_, *χ*_m_*T*, and *χ*_m_^−1^ (inset) collected in applied field of 1000 Oe for (a) **1** and (b) **2**. Red solid line represents best fits.

Magnetic susceptibility data for **1** and **2** were recorded in the temperature range of 2.0–300 K in an applied magnetic field of 1000 Oe. The *χ*_m_, *χ*_m_*T vs. T* plots for the complexes are shown in Fig. 3, where *χ*_m_ is the molar magnetic susceptibility. For supramolecular assemblies **1** and **2**, the values of *χ*_m_*T* at 300 K are 81.8 and 92.2 cm^3^ mol^−1^ K, respectively, but lower than that of expected for the sum of the Curie constants for 24 non-interacting Mn^II^ (*s* = 5/2) ions, with *g* = 2.00 (105.0 cm^3^ mol^−1^ K). Upon cooling, *χ*_m_*T* first gradually decreases to a value of 76.1 cm^3^ K mol^−1^ at 100 K, and then decreases more rapidly on further cooling to 27.3 cm^3^ K mol^−1^ at 2.0 K for **1**, however, *χ*_m_*T* decreases to the minimum value of 29.4 cm^3^ mol^−1^ K at 2.0 K for **2**, indicating antiferromagnetic coupling within the Mn^II^ ions. Above 50 K, the temperature dependence of *χ*_m_^−1^ obeys the Curie–Weiss law with *C* = 90.91 cm^3^ K mol^−1^ and *θ* = −12.8 K above 2.0 K for **1** and *C* = 102.88 cm^3^ mol^−1^ K and *θ* = −41.8 K for **2** (see Fig. 3, inset). The negative *θ* values confirm the antiferromagnetic coupling within the Mn^II^ ions and the antiferromagnetic coupling in **2** is stronger than that in **1**. Furthermore, the shapes of the *M*/*H* plots are quite like that of the antiferromagnet, in which the *M* values increase rapidly at low fields, with no obvious saturation observed up to 70 kOe (Fig. S11 and 12†).

Water oxidation (WO, 2H_2_O → O_2_ + 4H^+^ + 4e^−^) is regarded as a key half-reaction for solar fuel production.^39^ The rational design and synthesis of cheap, efficient and stable water-oxidising catalysts are significant challenges in science and technology.^40^ In nature, the oxygen-evolving complex (OEC, a CaMn_4_O_5_ cluster) in photosystem II (PS II) can efficiently oxidize water.^41^ It has been shown that the Mn^IV^–O–Mn^III^–H_2_O motif plays a crucial role in the activity of the OEC and its mimics.^42^ Inspired by the OEC, several Mn clusters have been used as structural mimics. In particular, the presence of high oxidation state +3 and +4 Mn ions and four water binding sites have been applied for electrocatalytic oxidation of water, examples such as Mn_12_O_12_(OAc)_16−*x*_L_*x*_(H_2_O)_4_ (L = acetate, benzoate, benzenesulfonate, diphenylphosphonate, and dichloroacetate).^43–45^ However, the catalytic activity of these biomimetic Mn-based clusters for water oxidation was shown to be hindered by either high overpotentials (ranging from 640–820 mV) or low structural stability.^40^ Kinetically and thermodynamically very stable Mn clusters assembled with exclusively Mn^II^ ions may solve one of such problem even though a series of mononuclear manganese complexes [(Py_2_NR_2_)Mn^II^(H_2_O)_2_]^2+^ (R = H, Me, *t*Bu) were reported to be active in electrocatalytic water oxidation with an relatively high overpotential of approximately 800 mV (FTO working electrode).^34^ However, to the best of our knowledge it remains unknown whether polynuclear Mn^II^ clusters are capable of being highly active with respect to water oxidation.

**Fig. 4:**
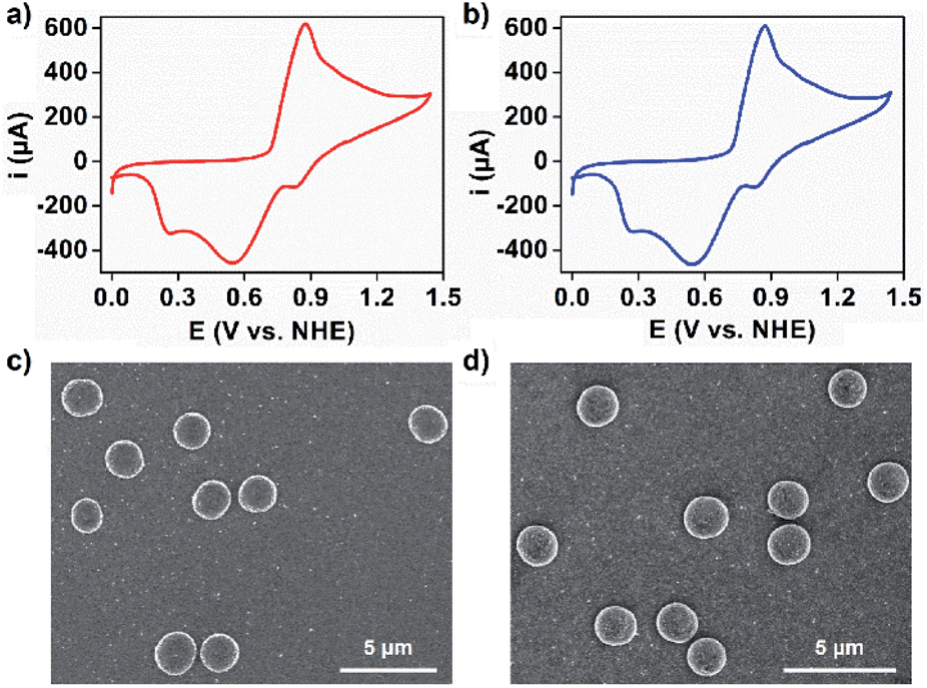
Cyclic voltammograms (CVs) of (a) **1** and (b) **2** (0.5 mM) in 0.1 M acetate buffer at pH 6.07 using an FTO (*S* = 1 cm^2^) working electrode. Scan rate is 50 mV s^−1^. SEM images of hierarchical micron spheroids formed from an aqueous acetate buffer of (c) **1** and (d) **2**.

This long-standing question has been examined with **1** and **2** using electrochemical techniques. Crystals of **1** and **2** were dissolved in 0.1 M aqueous acetate buffer at pH 6.07 *via* sonochemistry, the pH at which the OEC within PSII shows optimal catalytic performance.^46^ The resulting solutions of **1** and **2** were subjected to UV-Vis spectroscopy and showed two broad absorption bands at around *λ*_max_ = 262 and 315 nm for **1** and *λ*_max_ = 260 and 312 nm for **2**, which can be assigned to the π–π* transition and ligand-to-metal charge transfer transition, respectively (Fig. S13†). The redox peaks associated with manganese of **1** and **2** in aqueous acetate buffer have been detected *via* cyclic voltammetry (Fig. 4a, b and S14†). These corresponded to the oxidation of Mn^2+^ to Mn^4+^ (*E* = 0.87 V) and the reduction of Mn^4+^ to Mn^3+^ (*E* = 0.83 V), Mn^4+^ to Mn^2+^ (*E* = 0.55 V), and Mn^3+^ to Mn^2+^ (*E* = 0.26 V).^47^ The solution stability of the coordination structures was investigated using dynamic light scattering (DLS) techniques. It was shown that sonication of these solutions resulted in the formation of species in the size range of 2–3 nm, corresponding to the molecular hydrodynamic diameter of discrete MONCs (Fig. S15†),^13^ and implying that HSSs converted into discrete MONCs; we envisage that some metal-coordinated hydroxyl groups of PgC_3_OH moieties on axial positions may be displaced by water molecules. Interestingly, upon evaporation of an aqueous acetate buffer solution of **1** and **2**, spherical, micron-scale metallosuperstructures were observed by SEM (Fig. 4c, d and S16†). FT-IR and small angle X-ray scattering studies further supported that they were composed of many MONC subunits (Fig. S17 and 18†). We propose that the hierarchical metal–organic micron spheriods (MOMSs) may be stabilized by a large number of van der Waals interactions between neighboring alkyl chains and hydrophilic regions of the discrete MONCs.

**Fig. 5:**
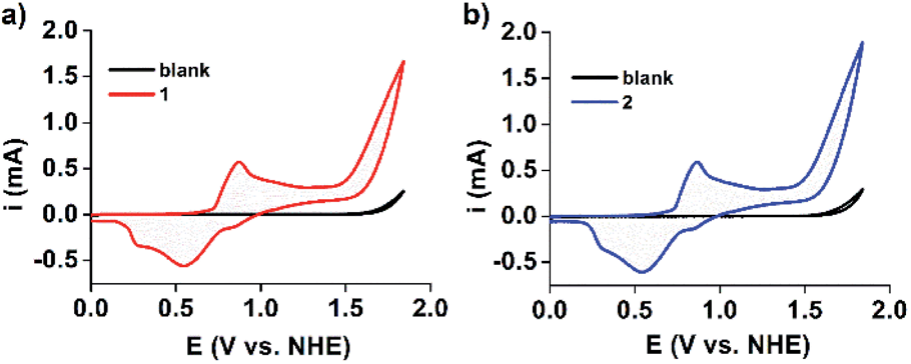
CV scans of (a) **1** and (b) **2** (0.5 mM, 50 mV s^−1^ scan rate) in 0.1 M acetate buffer at pH 6.07. For comparison, CVs of the blank buffer are also shown. FTO (*S* = 1 cm^2^) was used as the working electrode.

Furthermore, cyclic voltammograms (CVs) clearly indicated that water oxidation can be catalyzed by both **1** and **2** (Fig. 5).^43,47^ Water oxidation occurs at an exceptionally low overpotential of only 368 mV. This is higher than that of the current state-of-art Ru-bda complex (bda = 2,2′-bipyridine-6,6′-dicarboxylate, 180 mV at pH 7), illustrating that there is still room for further improvements.^48^ Continuous CV scan experiments and bulk electrolysis of **1** and **2** demonstrated that these electrocatalysts have high catalytic activity and stability toward water oxidation (Fig. S19 and 20†). UV-Vis and DLS measurements taken after electrolysis of **1** and **2** showed that the waves and particle size are retained (Fig. S21 and 22†). Moreover, the MOMSs re-formed and could be detected upon evaporation of the catalyst solution in subsequent SEM studies (Fig. S23†). Collectively, these measurements suggest that the MONC subunit is a homogeneous water oxidation electrocatalyst. This result may thus provide a new strategy for the design and synthesis of cheap, efficient and stable water-oxidizing catalysts since it first suggests that soluble Mn^II^ clusters may be used to effectively facilitate the oxidation of water, despite the enormous efforts made to mimic the CaMn_4_O_5_ cluster to date. We further envision that improvements of catalyst stability and activity may be possible. This may be achieved through (for example) attaching appropriate axial ligands to the constituent metal ions, or functionalizing alkyl chains on the MONC surface. In addition, other soluble metal-seamed dimeric or hexameric MONCs, such as those formed with Fe^II^, Co^II^ and Cu^II^ ions, are also promising with regard to electrocatalytic water oxidation.^36^

## Conclusions

In summary, we have developed a new strategy for the rational construction of HSSs using biomimetic self-assembly as the synthetic methodology. Akin to the self-assembly behaviours of protein subunits with redox-active metal ions, the assembly of these sophisticated supramolecular architectures has been accomplished by employing proline molecules as molecular chaperones to selectively insert redox-active Mn^II^ ions into the coordination sites of a pre-assembled ONC skeleton, which further directs the Mn^II^-seamed MONC subunits to fold and assemble into more complex HSSs across different dimensionality. This is achieved *via* control of both interior and/or exterior surface properties of the MONC subunits, through coordination and host–guest chemistries, also allowing for the fine-tuning of magnetic properties. The catalytic activity and stability of 
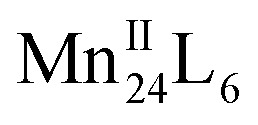
 MONCs toward homogeneous water oxidation at pH 6.07 with an exceptionally low overpotential of only 368 mV is noteworthy.

Overall, this approach represents an important advancement in supramolecular chemistry by design. Further efforts will be invested in the design and synthesis of extremely challenging and complex HSSs with other redox-active or coordinatively inert metal ions (*e.g.* Cr^II^/Cr^III^ and Fe^II^/Fe^III^), as well as inserting suitably functionalized guest molecules for potential application in the areas of molecular electronics/magnets and catalysis, all of which may be modulated by appropriate molecular chaperones. Finally, this strategy may be widely exploited in the rational design and synthesis of other metal–organic systems, such as metal–organic cages (MOCs), polyhedra (MOPs) or artificial metalloproteins, the properties and/or functions of which can be subsequently tailored accordingly.

## Conflicts of interest

The authors declare no conflict of interest.

## Supplementary Material

SC-011-D0SC05002D-s001

SC-011-D0SC05002D-s002
