# Supplementary material for: Controlled hierarchical self-assembly of networked coordination nanocapsules via the use of molecular chaperones
Source: Chem Sci. 2020 Oct 28;11(46):12547–52. doi: 10.1039/d0sc05002d (PMC8163202; doi:10.1039/d0sc05002d)
Supplement: SC-011-D0SC05002D-s001 [file SC-011-D0SC05002D-s001.pdf]

Electronic Supplementary Material (ESI) for Chemical Science.
This journal is © The Royal Society of Chemistry 2020

Supporting Information

Controlled hierarchical self-assembly of networked coordination nanocapsules via the use of molecular chaperones

Xiangquan Hu[1], Sisi Feng[2]*, Jialei Du[3]*, Li Shao[1], Jinxin Lang[4], Chen Zhang[5], Steven P. Kelley[1], Jian Lin[6], Scott J. Dalgarno[7], David A. Atwood[8] and Jerry L. Atwood[1]*

[1] Department of Chemistry, University of Missouri-Columbia, Columbia MO 65211, USA

[2] Key Laboratory of Chemical Biology and Molecular Engineering of Ministry of Education, Institute of Molecular Science, Shanxi University, Taiyuan 030006, P. R. China

[3] Institute for Advanced Interdisciplinary Research, University of Jinan, Jinan 250022, P. R. China

[4] School of Chemistry, Xi'an Jiaotong University, Xi'an 710049, P. R. China

[5] Department of Chemical and Biomolecular Engineering, North Carolina Sate University, Raleigh, North Carolina 27695, USA

[6] Department of Mechanical and Aerospace Engineering, University of Missouri-Columbia, Columbia MO 65211, USA

[7] Institute of Chemical Sciences, Heriot-Watt University, Riccarton, Edinburgh EH14 4AS, United Kingdom

[8] Department of Chemistry, University of Kentucky, Lexington, KY 40506, USA

*Corresponding author: ssfeng@sxu.edu.cn, ifc_dujl@ujn.edu.cn, atwoodj@missouri.edu

This file includes:

Figures S1 to S23

Tables S1 to S2

References 1 to 15

1. Materials and Methods.

All solvents and chemicals were purchased from Sigma-Aldrich or Fisher Laboratories and used without further purification. Notably, manganese (II) nitrate tetrahydrate crystals were stored in glovebox at 23°C. The solvents were dried by 3Å molecular sieves for uses of solvothermal synthesis. All combinations of PgC_3OH macrocycles and manganese salts were carried out using glovebox techniques at 23°C. All pH measurements were performed using a Thermo Scientific Orion Star A111 Benchtop pH Meter. Powder X-ray Diffraction data was collected on a Bruker Apex II CCD diffractometer at room temperature using Cu (Kα) radiation Inco-tech Microfocus II (1.5406Å). Powder X-ray diffraction was measured on a Bruker X8 Prospector single crystal X-ray diffractometer equipped with an IµS microfocus Cu-Kα X-ray source (λ = 1.54106 Å, power = 40 kV, 0.65 mA). Dry samples were hand-ground into powder and loaded directly into the tubing. Data collection was performed with the area detector and X-ray source fixed, and the tubing containing the sample at a 90° angle to the X-ray beam at a sample-to-detector distance of 8.00 cm. The samples were rotated 360° along the axis of the tubing during collection. Each data set was composed of a series of 2-minute long scans across the 2-theta range of 2.5 to 40°. Photographic data were reduced by integrating along a 77°-wide sector from 2.5 to 35° 2-theta in 0.02° slices along 2-theta. Small angle X-ray scattering analyses was characterized with Xenocs SAXS equipment. The experiment was conducted in 1200s, under a power of 50 kV, 0.6 mA, using a PILATUS 100K detector and the wavelength was 0.15148. Positive ion MALDI (Matrix-Assisted Laser Desorption Ionization) TOF (Time of Flight) mass spectrometer measured on a Bruker Autoflex Speed MALDI TOF MS using dithranol as the matrix. Samples in water was combined with methanol containing dithranol molecules. FT-IR spectra were recorded at room temperature using a Thermo Nicolet Avatar 360 FTIR Spectrometer in the 400–4000 cm^{-1} range. Elemental analysis (EA) was performed using a European A3000 Elemental Analyzer. Thermogravimetric analysis (TGA) was performed using a TA Instruments Q50 TGA, with a Pt sample pan under 40 mL min^{-1} nitrogen purge. The sample was heated from room temperature to 800 °C at the rate of 20 °C/min. Differential scanning calorimetry (DSC) was performed using a TA Instruments Q1000@Mfg-dsc, with an Al hermetic sample pan under 40 mL min^{-1} nitrogen purge. The sample was heated from 40 °C to 600 °C at the rate of 10 °C/min. X-ray photoelectron spectroscopy (XPS) spectra were recorded using a Thermo Fisher Scientific Escalab 250. UV-visible (UV-Vis) spectra were measured using a Varian 50 BIO spectrophotometer. Crystals of **1** and **2** was suspended in 0.1 M aqueous acetate buffer at pH 6.07. The mixture was sonicated for 15 min at 45 °C to yield a yellow solution. The solution was filtered using a Whatman Puradisc 30 syringe filters (pore size 0.2 µm) and then subjected to UV-Vis analysis. Corresponding UV-Vis samples in acetate buffer (filtered

solution) were subjected to Dynamic light scattering (DLS) analysis with BECKMAN COULTER DelsaTM Nano C particle analyser. Scanning electron microscopy (SEM) images were obtained in field emission scanning electron microscope (FESEM; MERLIN Compact, Carl Zeiss) at an acceleration voltage of 200 kV. Corresponding UV-Vis and DLS samples in acetate buffer (filtered solution) were drop-casted on a silicon wafer following naturally drying and then washed with Milli-Q ultrapure water.

Single crystal X-ray diffraction data for **1** were collected on a Bruker Apex II diffractometer equipped with a CCD area detector using Mo-Kα radiation from a fine-focus sealed source with a focusing collimator (Bruker Nano). Data for **2** were collected on Bruker D8 Venture diffractometer with a Photon 100 CMOS area detector using Mo-Kα radiation (λ = 0.71073 Å) from an IμS microfocus source (Bruker Nano, Inc., Madison, WI, USA). Crystals were cooled to 100 K under a cold stream of N_2 gas using a Cryostream 700 cryostat for **1** (Oxford Cryosystems) and a Cryostream 800 cryostat (Oxford Cryosystems, Oxford, UK) for **2**. Hemispheres of unique data were collected using strategies of scans about the phi and omega axes. The Apex3 software suite was used for data collection, unit cell determination, data reduction, scaling, and absorption correction.[1]

Compound **1** was solved and refined using SHELXL-2017[2] and SHELXT[3] as the interface. The ordered portion of the structure was refined anisotropically. Some of the propanol chains were so strongly affected by disorder that no clear interpretation of the difference map was possible; however each PgC_3OH moiety has at least 1 fully ordered propanol chain which confirms the identity of the moiety. For the disordered chains, the closest reasonable difference map peaks were refined as propanol chains using distance and angle restraints. Atoms that gave clearly unrealistic geometries or displacement parameters were not given any riding hydrogen atoms. These atoms were left in the structure to help make the disordered solvent calculation more accurate and allow better visualization of the disordered regions. Compound **1** also showed difference map peaks inside the MONCs which closely resembled the expected geometry of a proline ligand, but attempts to refine this ligand failed to converge with a realistic geometry. Ultimately the entire proline moiety was modeled as a rigid group using coordinates from a published structure with a similar conformation[4] and using a single parameter to describe the atomic displacements for all ring carbon atoms. This refinement revealed that the proline ring was disordered over two conformations, both of which could be modeled with distance and angle restraints. The difference map also indicated the presence of a second unique proline molecule in the cavity, but not all atoms could be located. This molecule was ultimately excluded from the model and treated with

a solvent mask. *Olex2* v. 1.3.0 was used for model building and as an interface for SHELX.[5] PLATON SQUEEZE was used to implement solvent masks.[6] Literature coordinates were obtained from the Cambridge Crystallographic Data Center using the database searching software ConQuest V. 2.0.5.[7]

Compound **2** was solved by isomorphous replacement. The coordinates of the isomorphous Mg^{2+} analog[8] were used as an initial model with all Mg sites replaced with Mn. The structure was refined to convergence by full matrix least squares refinement against F^2 using SHELXL-2017.[2] The diffraction data for the crystal was essentially negligible beyond 1.1 angstroms (R_{int} > 50% for the 1.44-1.39 angstrom shell; average I/sigma at 1.10 is approximately 0.40). Due to the lower data-to-parameters ratio of the Mn^{2+} model caused by the weak diffraction, some disordered lattice solvent molecules from the Mg^{2+} model were removed using a solvent mask. The converged model (after solvent masking) has a GooF near 1 and a reasonably smooth residual difference map, both indicate the coordinates from the Mg^{2+}model agree well with the Mn^{2+} data. The presence of two lattice acetonitrile molecules from the Mg^{2+}model that refine well further supports that the packing in these two structures is almost identical. H atoms could not be located for O-H groups and were left out of the model but included in the formula. The identities of the axial ligands bound the Mn ions are uncertain from the X-ray diffraction data, so only the coordinating O atoms were included in the formula. The formula assumes that Mn^{2+} ions are charge balanced by deprotonated phenolic O-H groups.

2. Synthesis of *C*-propan-3-ol pyrogallol[4]arene (PgC_3OH).

C-propan-3-ol pyrogallol[4]arene **1** was prepared by a condensation reaction of pyrogallol and 2,3-Dihydrofuran catalyzed by concentrated hydrochloric acid.[9] 2,3-Dihydrofuran (6.05 mL, 0.08 mol), and pyrogallol (0.08 mmol, 10 g) were mixed in ethanol (40 mL) followed by the addition of 3.5 ml of concentrated HCl. Thereafter, the mixture was heated to reflux at 110 °C for 24 h. After cooling down, the precipitate was filtered, washed with cold ethanol and dried in vacuum. 5.4 g of white solid was collected as the final product. Yield is 34.8 %.

3. Investigations of reaction conditions of HSSs.

PgC_3OH (0.2 mmol), a source of Mn^{II} ions, $Mn(NO_3)_2$, $MnSO_4$ and $MnCl_2$ (0.8 mmol) and NaOMe (0.6 mmol) or triethylamine (112 µl, 0.8 mmol) were combined in DCM/MeOH (10 mL each). Upon slow evaporation of the mother liquor over six weeks, no MONCs-based crystals/precipitates were observed, but some dark insoluble MnO_2 was found to precipitate when utilizing $Mn(NO_3)_2$ as the source of Mn^{II}

ions. The formation of MnO_2 was confirmed by reaction with HCl which would lead to the formation of $MnCl_2$ crystals. This indicates that *in-situ* redox reactions may prevent the formation of HSSs, and that the formation of these highly intricate structures is unfavorable under ambient conditions, owing to their high structural strength. Efforts were then made to determine whether the formation of HSSs could be facilitated under solvothermal conditions. PgC_3OH (0.1 mmol, 78.4 mg) and manganese slats, including $Mn(NO_3)_2·4H_2O$, $MnSO_4·H_2O$ and $Mn(Cl)_2$ (0.4 mmol, 100.4 mg, 67.6 mg, 50.3 mg, respectively) were dissolved 1.5 mL of N,N-dimethylformamide (DMF), 1.5 mL of acetonitrile (CH_3CN) and 0.1 mL water, followed by the addition of 16 mg sodium methoxide (0.3 mmol). The mixture was sonicated for 30 min at 45 °C to yield a dark brown solution (final pH=4.47), and then heated at 80 °C for 12 hours. Dark insoluble MnO_2 precipitates and colorless block-like crystals of a well-known $Mn_3(HCOO)_6]$· DMF metal-organic frameworks were obtained,[10] yield is 8 mg. The formation of either MnO_2 precipitation or a well-known $[Mn_3(HCOO)_6]$ metal-organic framework, implying that they compete with the selective assembly of Mn^{II}-seamed MONCs and HSSs.

4. Preparation and characterization of the HSSs crystals 1 and 2

Preparation of **1**.

PgC_3OH (0.1 mmol, 78.4 mg) and $Mn(NO_3)_2·4H_2O$ (0.4 mmol, 100.4 mg) were dissolved 1.5 mL of N,N-dimethylformamide (DMF), 1.5 mL of acetonitrile (CH_3CN) and 0.1 mL water, followed by the addition of 36 mg L-proline (0.3 mmol). The mixture was sonicated for 30 min at 45 °C to yield a dark brown solution (final pH=4.13), and then heated at 80 °C for 12 hours. Large yellow block crystals were then formed and collected for single crystal X-ray analysis. Unit cells of several crystals (protected in oil) were checked in order to establish sample homogeneity. Yield: 68 mg=58% (with respect to PgC_3OH ligand). The crystals are sensitive to loss of solvent and exposure to air, and thus acquiring a suitable X-ray diffraction powder patterns were not successful. MALDI-TOF-MS: $[Mn_{24}(PgC_3OH)_6(H_2O)_{24}(Proline)_2]$, ideal $C_{250}H_{332}O_{140}N_2Mn_{24}$, m/z = 6945; found m/z = 6600-7500 Da. Elemental analysis (%): $[Mn_{24}(PgC_3OH)_6(H_2O)_{24}(Proline)_2]·16(DMF)·8H_2O·2CH_3CN$, ideal $C_{302}H_{446}O_{164}N_{20}Mn_{24}$: C, 43.45; H, 5.35; N, 3.36; Found: C, 43.27; H, 5.42; N, 3.45.

Crystallographic data for **1** (CCDC: 1981690): monoclinic, space group P21/n (No. 14), a = 22.3705 (17), b = 32.550 (2), c = 22.5838 (17) Å, β = 95.026 (2)°, V = 16381 (2) Å^3, Z = 2, Dc = 1.356 g cm^{-3}, F_{000} = 6824, Bruker APEX II area detector, MoKα radiation, λ = 0.71073 Å, T = 100.0 K, $2\theta_{max}$ = 46.8°, 223225 reflections collected, 23785 unique (R_{int} = 0.0810). Final GooF = 1.678, R1 = 0.1318, wR2 = 0.3823, R

indices based on 14496 reflections with I > 2σ (I) (refinement on F^2), 1581 parameters, 1534 restraints. Lp and absorption corrections applied, μ = 0.974 mm^{-1}.

Preparation of **2**.

PgC_3OH (0.1 mmol, 78.4 mg) and $Mn(NO_3)_2·4H_2O$ (0.4 mmol, 100.4 mg) were dissolved 1 mL of N,N-dimethylformamide (DMF) and 2 mL of acetonitrile (CH_3CN) with the addition of 0.5 ml of water and 36 mg L-proline (0.3 mmol) in a 4 ml glass vial (final pH=4.2). The mixture was sonicated for 30 min at 45 °C to yield a dark brown solution, and then heated at 130 °C for 12 hours. Large orange block crystals were then formed and collected for single crystal X-ray analysis. Unit cells of several crystals were checked in order to establish sample homogeneity. Yield: 70 mg=61% (with respect to PgC_3OH ligand). MALDI-TOF-MS: $[Mn_{24}(PgC_3OH)_6(H_2O)_{44}]$, ideal $C_{240}H_{324}O_{140}Mn_{24}$, m/z = 6765; found m/z = 6500-7700 Da. Elemental analysis (%): $[Mn_{24}(PgC_3OH)_6(H_2O)_{44}]·14(DMF)·10H_2O·2CH_3CN$, ideal $C_{290}H_{454}O_{164}N_{18}Mn_{24}$: C, 42.83; H, 5.58; N, 3.11. Found: C, 42.67; H, 5.27; N, 3.16.

Crystallographic data for **2** (CCDC: 1981691): orthorhombic, space group Pccn (No. 56), a = 37.471 (5), b = 39.182 (5), c = 25.613 (4) Å, V = 37604 (9) $Å^3$, Z = 4, Dc = 1.209 g cm^{-3}, F_{000} = 13952, Bruker VENTURE CMOS area detector, MoKα radiation, λ = 0.71073 Å, T = 100.0 K, $2θ_{max}$ = 37.8°, 164260 reflections collected, 14844 unique (R_{int} = 0.2472). Final GooF = 1.013, R1 = 0.0858, wR2 = 0.2134, R indices based on 7398 reflections with I > 2σ (I) (refinement on F^2), 1843 parameters, 1961 restraints. Lp and absorption corrections applied, μ = 0.852 mm^{-1}.

Manganese Bond-Valence Sum (BVS) Analysis

Table S1. Bond-valence sum analysis for Mn1-Mn12 in **1** and their corresponding oxidation state assignments.[11]

Identity	Calculated Value	Assignment
Mn1	2.179	+2
Mn2	2.147	+2
Mn3	2.159	+2
Mn4	2.076	+2
Mn5	1.832	+2
Mn6	2.118	+2
Mn7	2.048	+2
Mn8	1.824	+2
Mn9	2.171	+2
Mn10	2.222	+2

Mn11	2.046	+2
Mn12	2.384	+2

Table S2. Bond-valence sum analysis for Mn1-Mn12 in **2** and their corresponding oxidation state assignments.

Identity	Calculated Value	Assignment
Mn1	2.167	+2
Mn2	2.199	+2
Mn3	2.066	+2
Mn4	2.404	+2
Mn5	2.320	+2
Mn6	2.251	+2
Mn7	2.207	+2
Mn8	2.331	+2
Mn9	2.265	+2
Mn10	2.192	+2
Mn11	2.234	+2
Mn12	2.352	+2

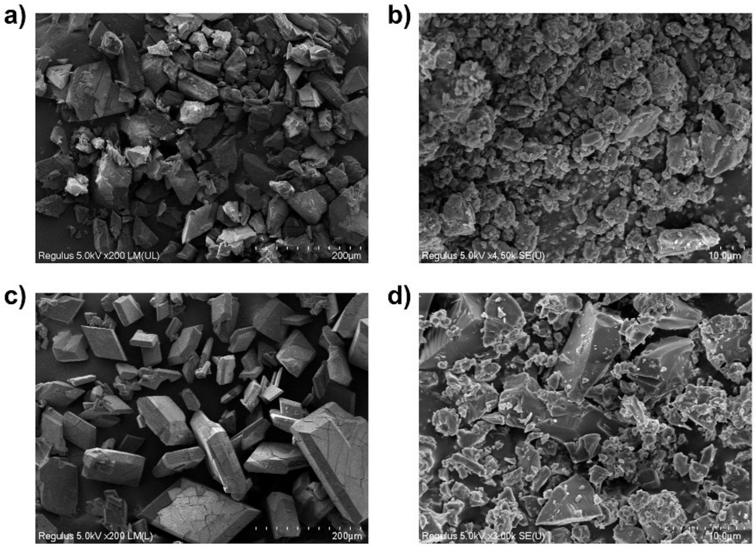

Figure S1. SEM images of **1** (a), (b) and **2** (c), (b).

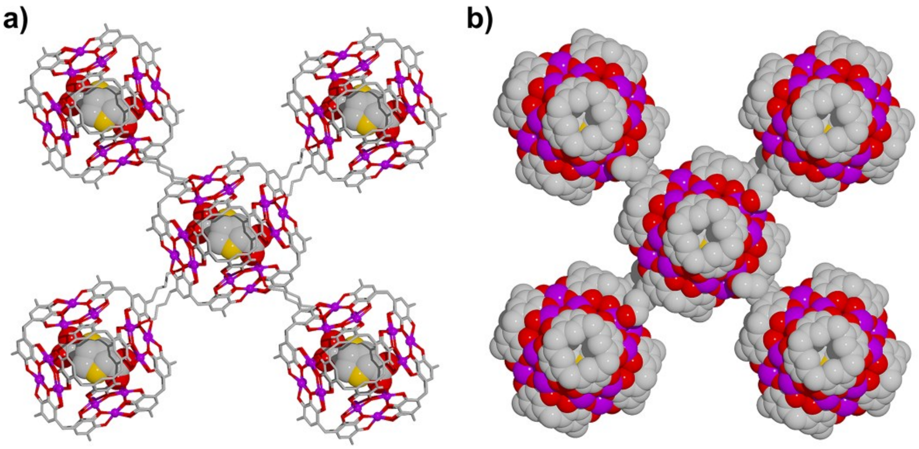

Figure S2. Side views of a) stick and b) space-filling model and of **1.** Color codes: manganese atoms are green; carbon atoms are yellow; oxygen atoms are red; nitrogen atoms are orange. Hydrogen atoms, axial water ligands, and hydroxyl alkyl chains of PgC_3OH were removed for clarity. The proline molecule itself can form stable metal complexes with Mn^{II}, this may be attributed to it contains both carboxyl groups and N-heterocycle substituents belonging to amino acid residues of manganese proteins, which have been shown high affinity for coordinating and stabilizing Mn^{II} ions.[12]

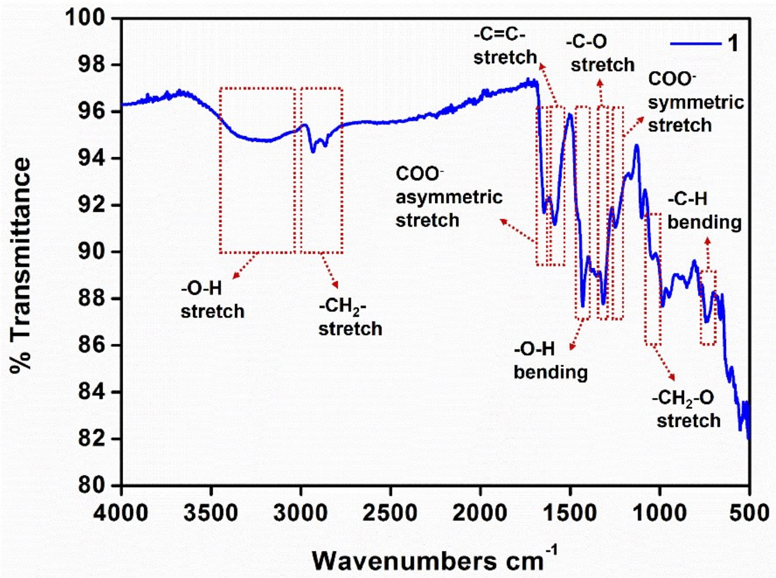

Figure S3. FTIR spectra of **1**. The absorption peaks of the stretching vibration bands derived from -CH2-, -COO-, -C-O, -OH and benzene ring on proline and PgC_3OH were observed in FTIR spectra.

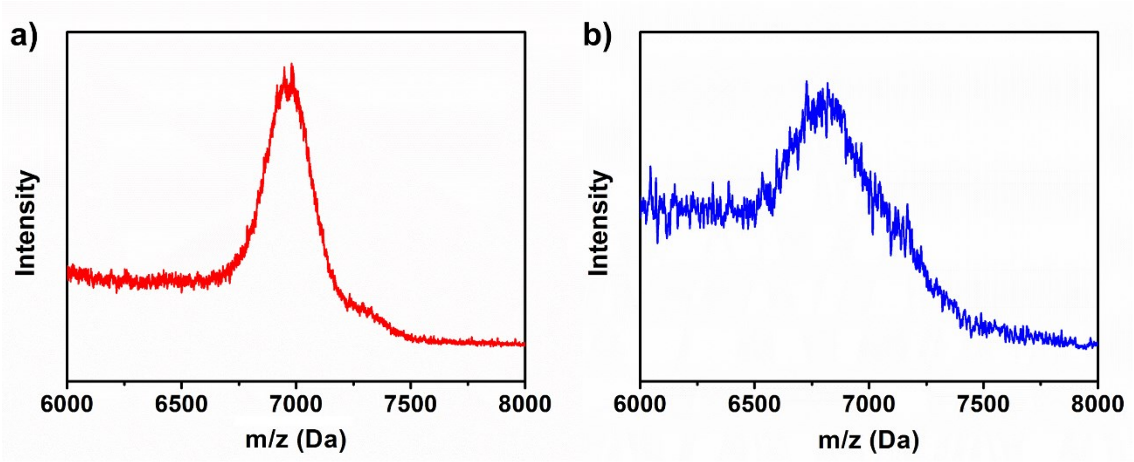

Figure S4. MALDI-TOF spectrum for **1** (a) and **2** (b) in methanol/water (1:1). The spectrum was obtained by using dithranol as the matrix.

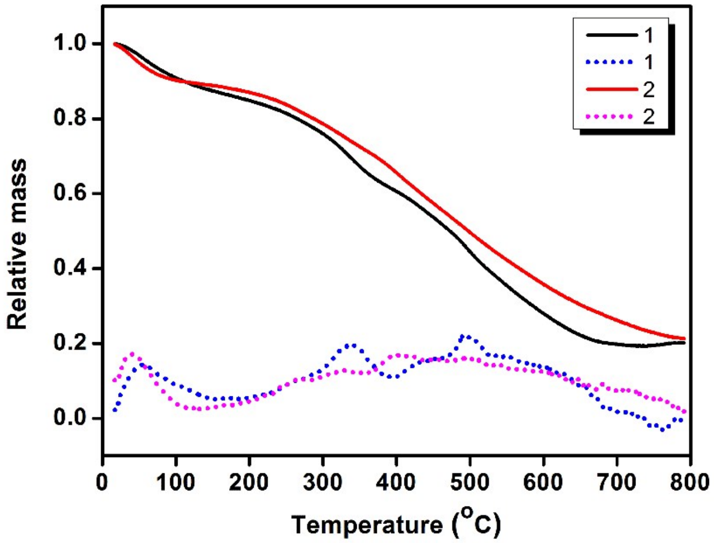

Figure S5. Thermogravimetric Analysis (TGA) Graphs (solid lines) and its first derivative value (dash lines) of **1** and **2**, showing three major distinct stages during gradual heating of a dried sample. 1) loss of trapped solvent molecules at around 100 °C; 2) breaking of crystal lattice, release of axial ligands and encapsulated solvent molecules within MONC subunits at around 320 °C; and 3) decomposition of the MONC subunits at around 500 °C.

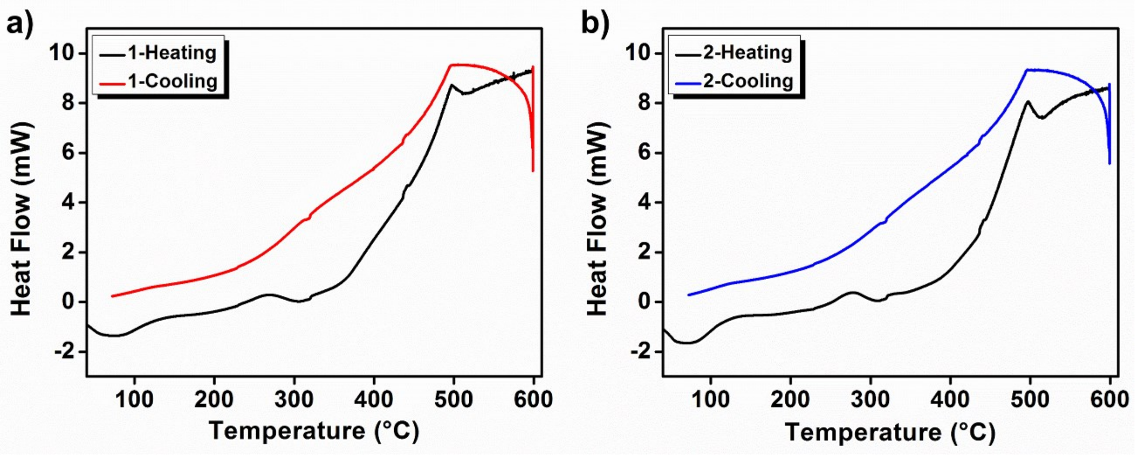

Figure S6. Differential scanning calorimetry (DSC) Graphs of (a) **1** and (b) **2**, indicating that solvent loss begins at around 40 °C (negative slope at the very beginning indicates an endothermic process). This continues until about 320 °C when the heat flow goes from negative (endothermic) to positive (exothermic), which suggests that at this point the crystal is reacting chemically. By 500 °C the reaction is complete.

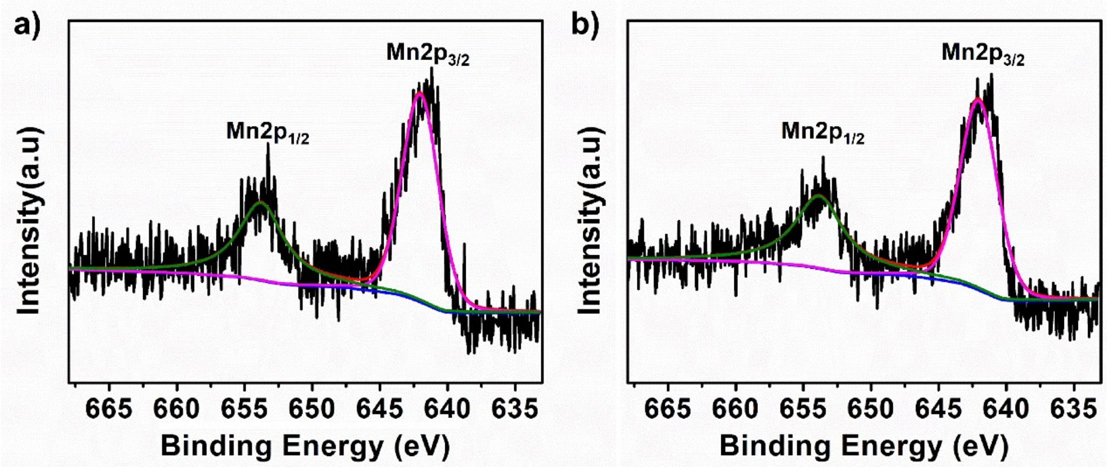

Figure S7. Mn2p XPS spectrum of **1** (a) and **2** (b). The XPS spectrum of **1** and **2** only illustrated the presence of Mn^{II} ions with two sharp Mn $2p_{3/2}$ and Mn $2p_{1/2}$ signals at 642.0 eV and 653.8 eV for **1**, 642.1 eV and 653.9 eV for **2**, respectively.[13]

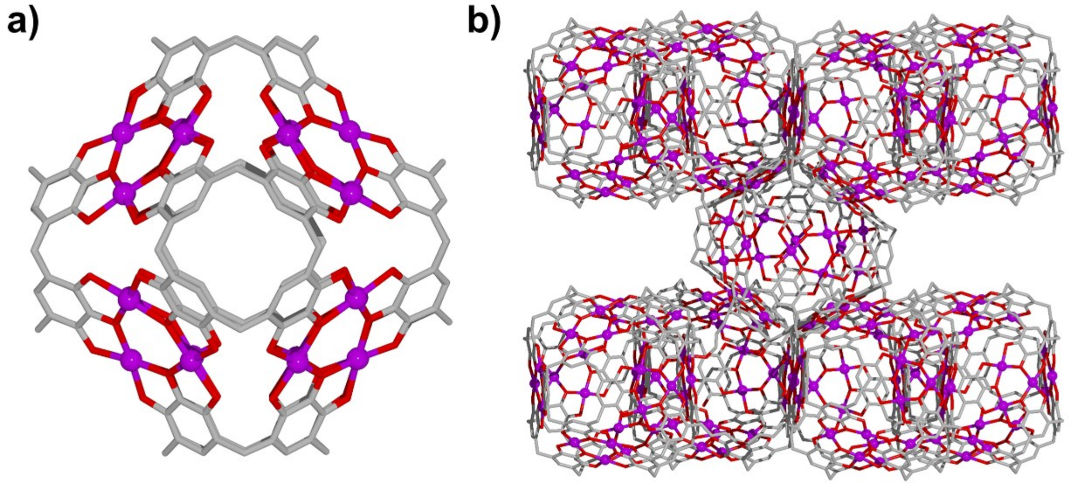

Figure S8. Crystal structure of a) MONC subunits (secondary structure) and b) supramolecular nanocubes (tertiary structure) within **2**. Color codes: manganese (purple), carbon (grey), oxygen (red). Hydrogen atoms, axial ligands and hydroxyl tail alkyl chains not involved in metal–ligand coordination to adjacent MONC subunits were removed for clarity. Proline molecules could not be explicitly modeled in the crystal structure, but they are essential for the synthesis.

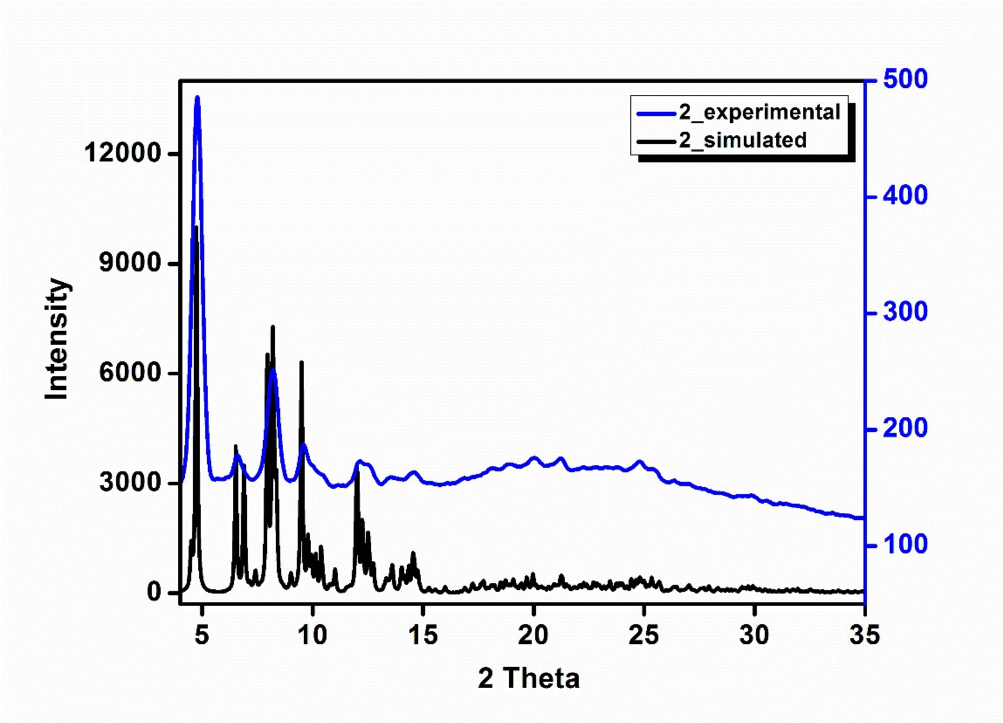

Figure S9. Simulated and experimental powder X-ray Diffraction patterns of **2**, showing high crystal stability, which the PXRD pattern of **2** matches well with the simulated pattern. This observation suggests that, construction of 3D frameworks using these MONCs as subunits may be an alternative way to characterize and study their structures.

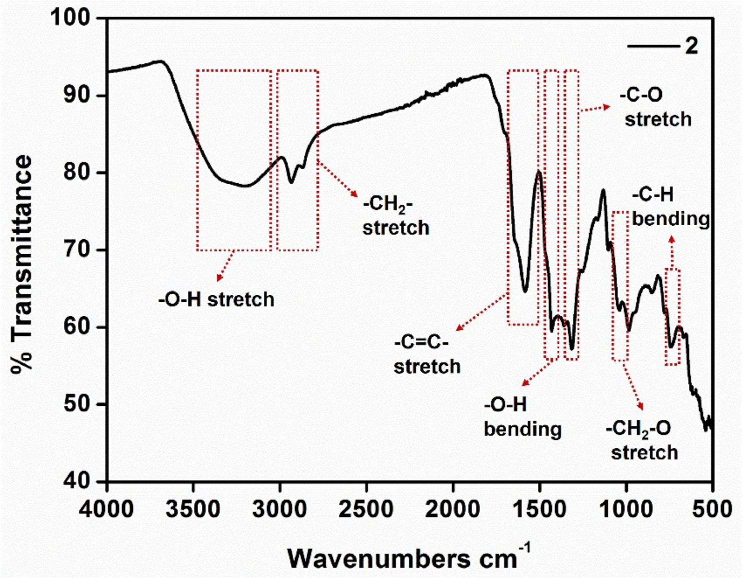

Figure S10. FTIR spectra of solid **2**. The absorption peaks of the stretching vibration bands derived from -CH2-, -CO, -OH and benzene ring on PgC_3OH were observed in FTIR spectra.

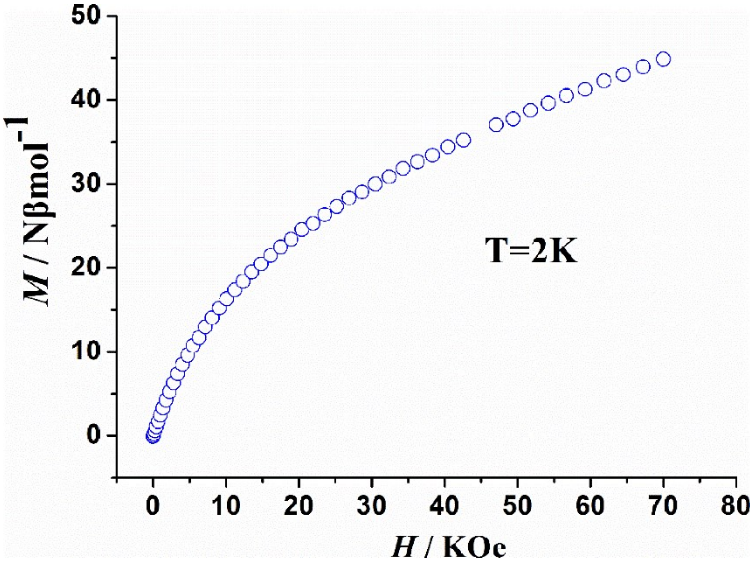

Figure S11. Magnetization analysis for compound **1**. Magnetization (*M*) vs. field (*H*) for **1** in the indicated field.

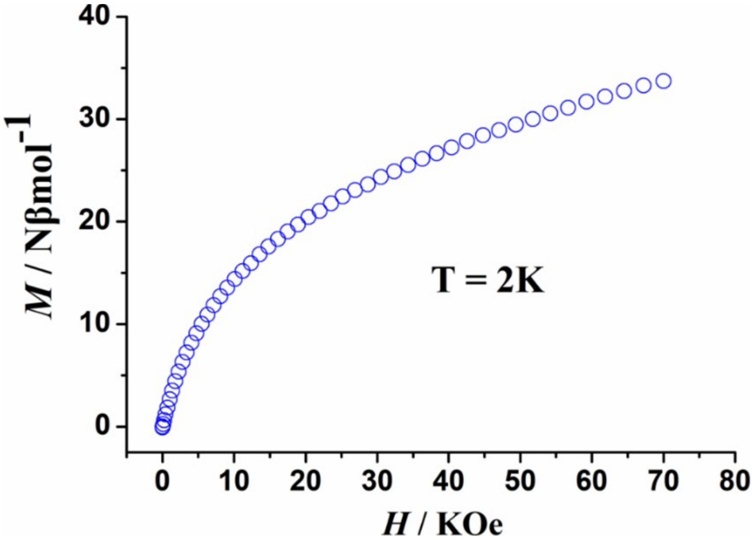

Figure S12. Magnetization analysis for compound **2**. Magnetization (*M*) vs. field (*H*) for **2** in the indicated field.

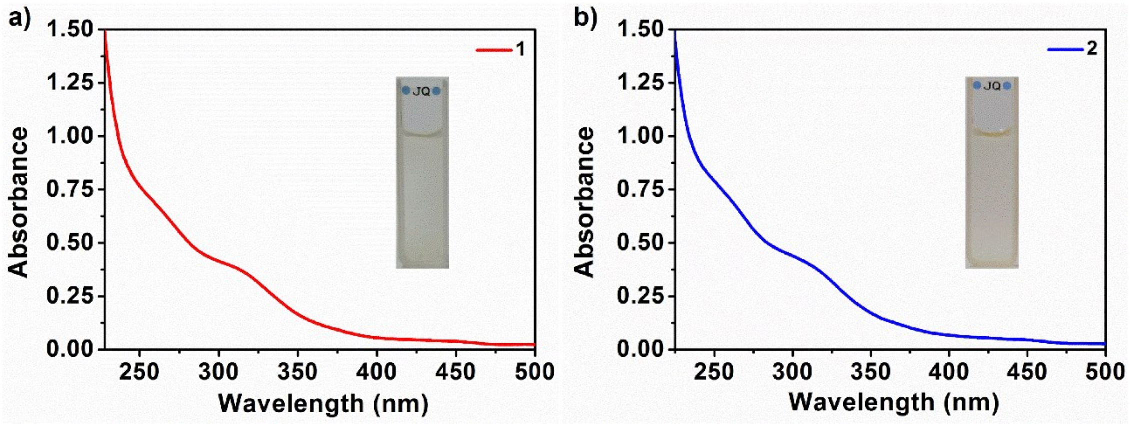

Figure S13. UV-vis spectra and photograph of **1** (a) and **2** (b) in 0.1 M acetate buffer at pH 6.07.

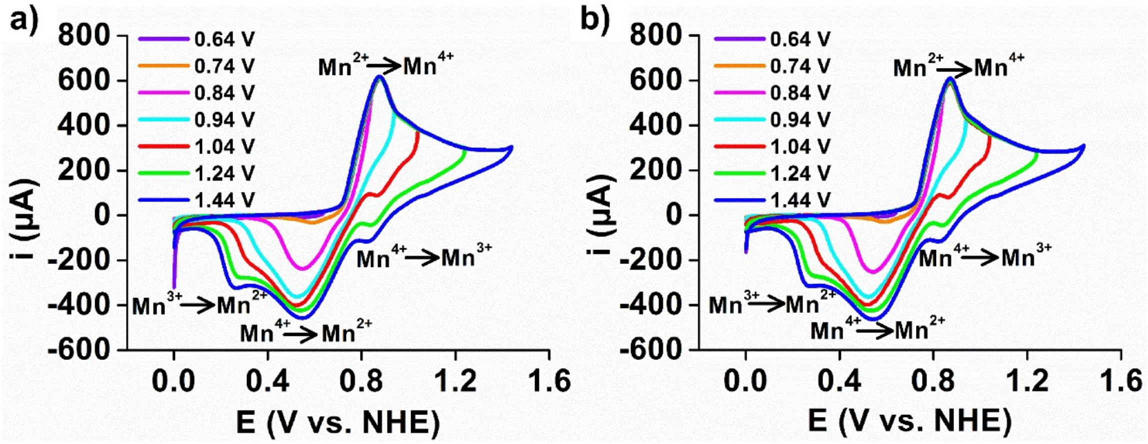

Figure S14. CV scans of **1** (a) and **2** (b) (0.5 mM, 50 mV s^{-1} scan rate) in 0.1 M acetate buffer at pH 6.07 taken at different potential ranges.

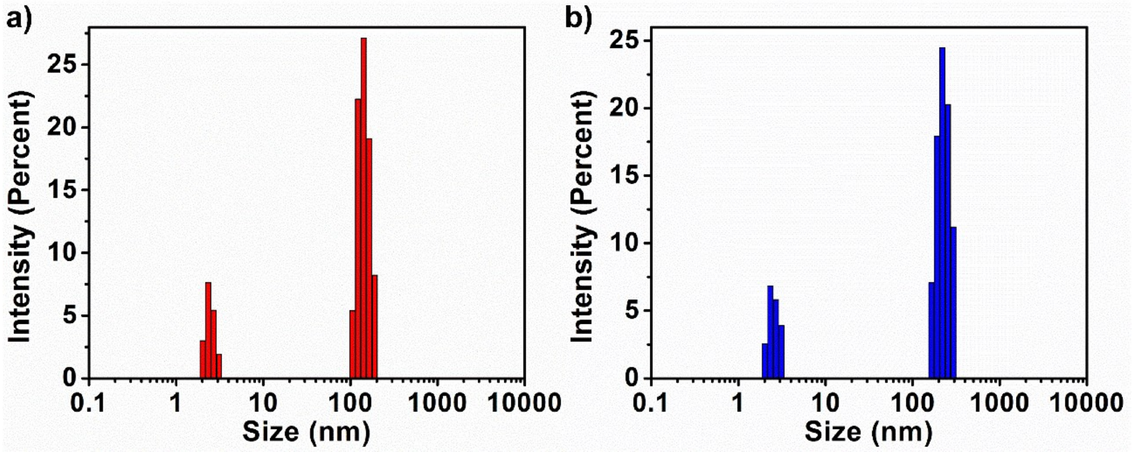

Figure S15. DLS measurements for **1** (a) and **2** (b) 0.1 M acetate buffer at pH 6.07 (0.5 mM). The DLS analysis of **1** and **2** indicated the existence of individual MONCs (molecular hydrodynamic diameter: 2-3 nm). This observation suggests that, in aqueous acetate buffer, the HSSs converted into discrete MONCs and these MONCs tend to exist as large aggregates.

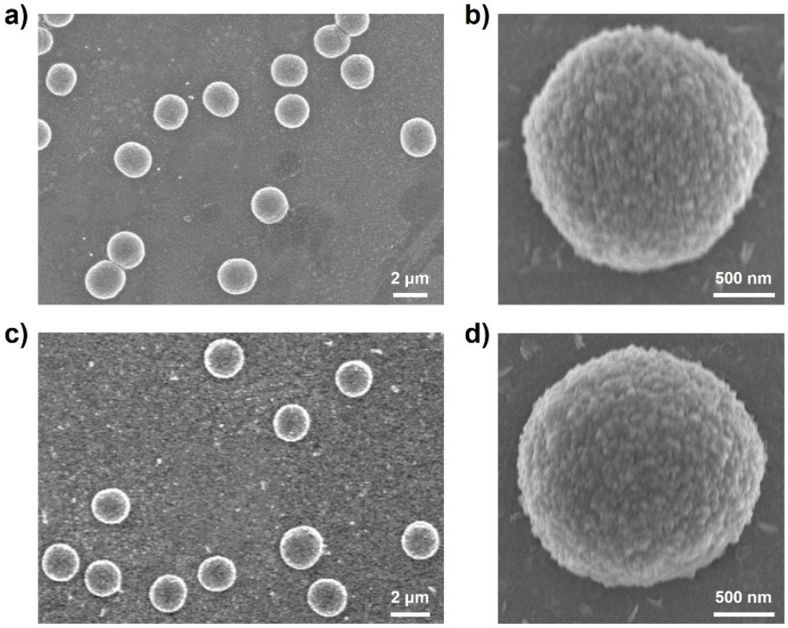

Figure S16. SEM images of micron spheres of 1 (a, b) and 2 (c, d) in 0.1 M acetate buffer at pH 6.07 (0.5 mM).

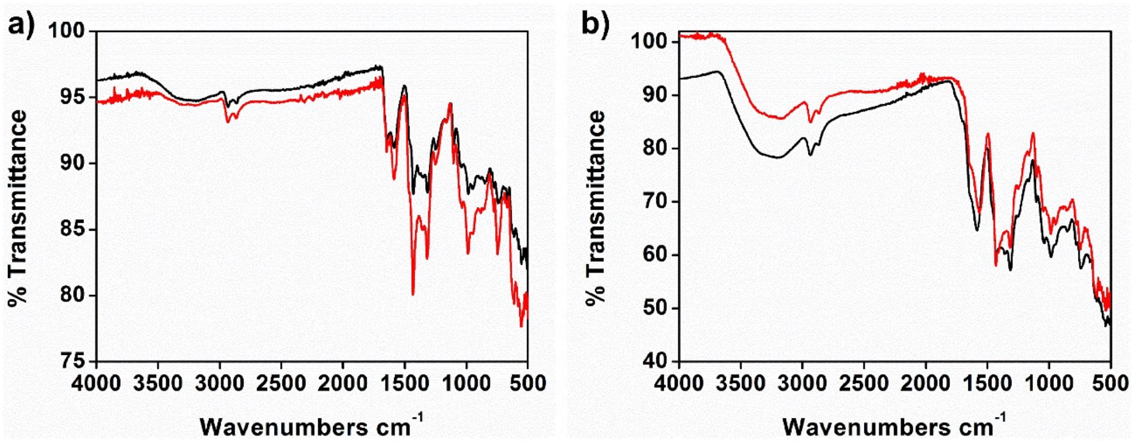

Figure S17. FT-IR spectra of **1** (a) and **2** (b) before (black lines) and after (red lines) dissolved in 0.1 M acetate buffer at pH 6.07 for two weeks (0.5 mM). These results indicate the existence of individual MONCs.

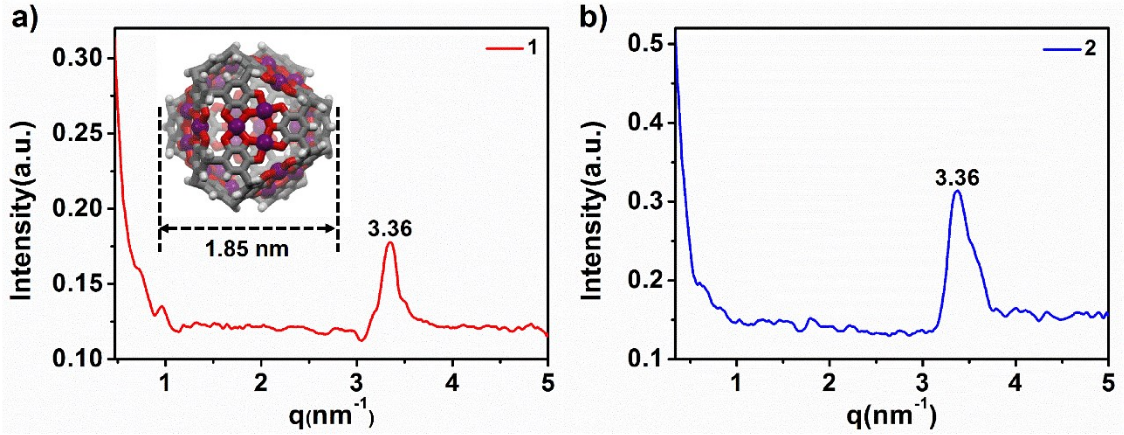

Figure S18. Small-Angle X-ray Scattering (SAXS) analysis of **1** (a) and **2** (b) after dissolved in 0.1 M acetate buffer at pH 6.07 for two weeks (0.5 mM). The broad peaks at scattering vector q =3.36 nm^{-1}, correspond to the spherical nanostructure with a diameter of 1.87 nm, which were assigned to the size of individual MONCs.[15]

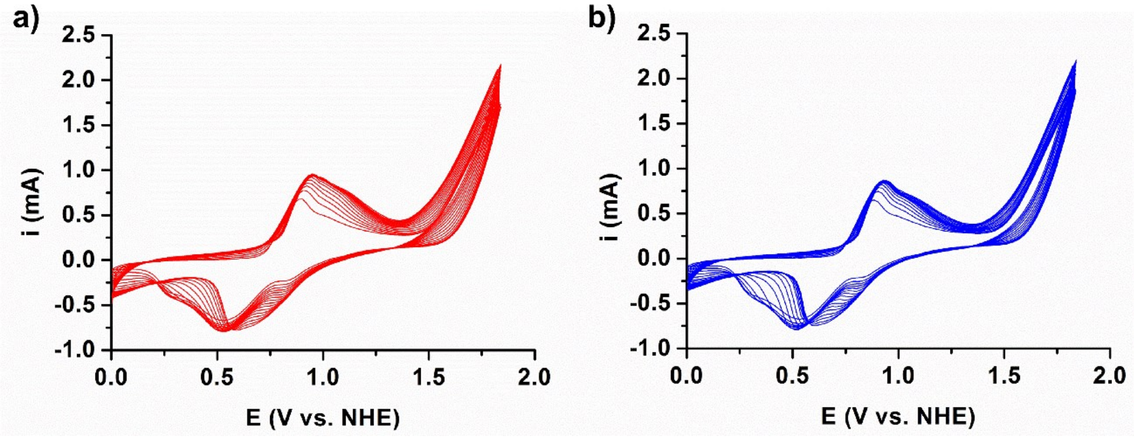

Figure S19. Continuous 30 CVs of **1** (a) and **2** (b) (0.5 mM, 50 mV s^{-1} scan rate) in 0.1 M acetate buffer at pH 6.07 using FTO as the working electrode. The catalytic current does not increase over successive cyclic voltammetric (CV) scans. A crossover profile with a re-reduction wave in the reverse CV scan was not observed. These observations suggest that MONC subunit is a homogeneous catalyst for water oxidation.[14]

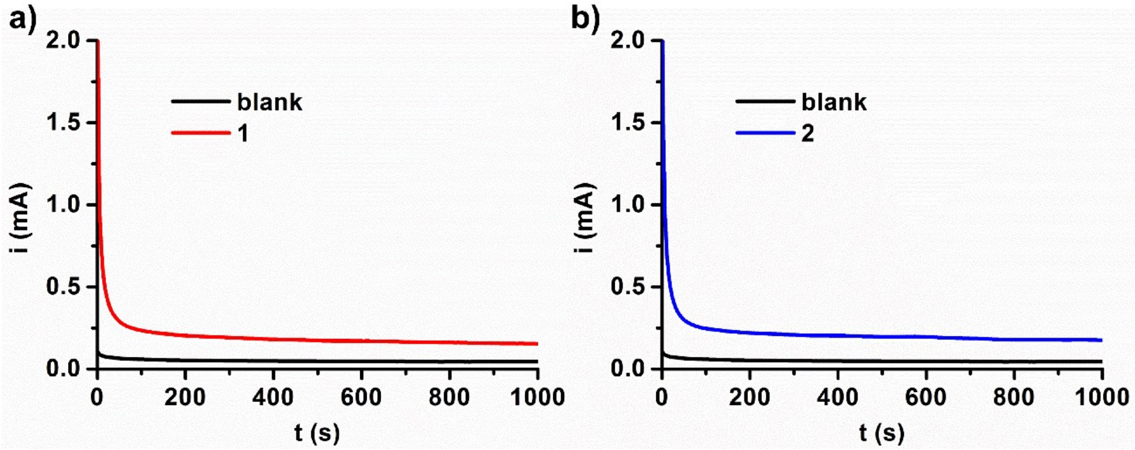

Figure S20. Bulk electrolysis at 1.79 V vs. NHE of 1 mM **1** (a) and **2** (b) in 0.1 M acetate buffer at pH 6.07 using FTO as the working electrode. For comparison, bulk electrolysis of the blank buffer is also performed. The catalytic current does not increase, as would be expected for a heterogeneous catalytic system.

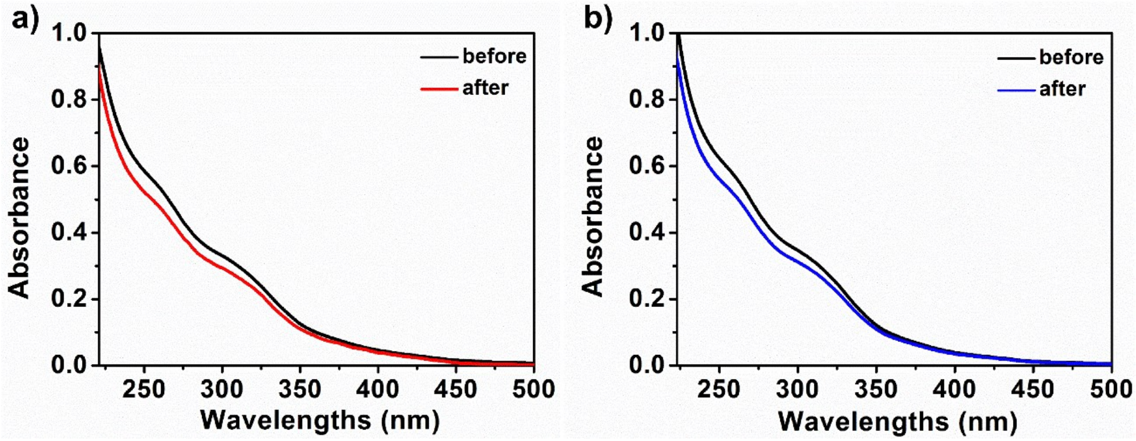

Figure S21. UV-vis spectra of **1** (a) and **2** (b) in 0.1 M acetate buffer at pH 6.07 after electrolysis. No new bands are observed, suggesting that the formation of new species is unlikely.

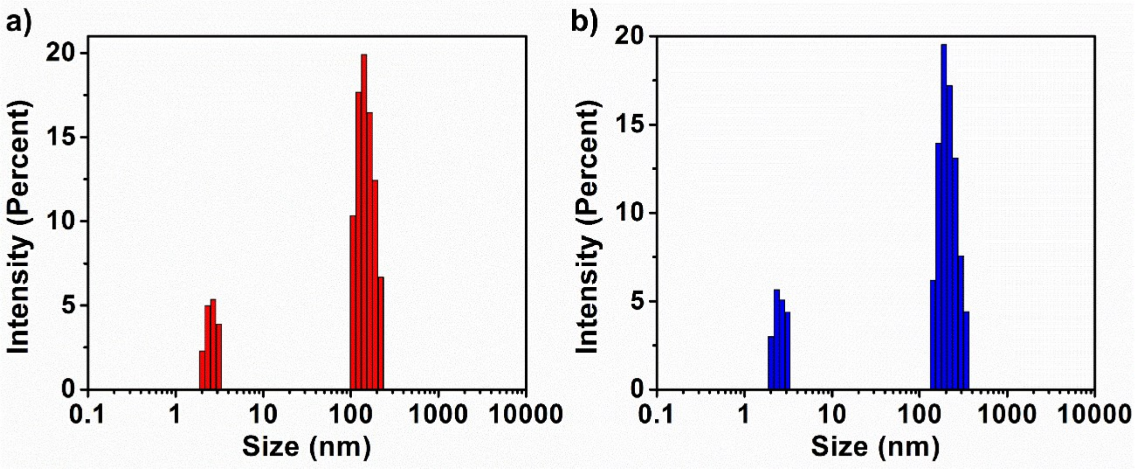

Figure S22. DLS measurements for **1** (a) and **2** (b) in 0.1 M acetate buffer at pH 6.07 after electrolysis.

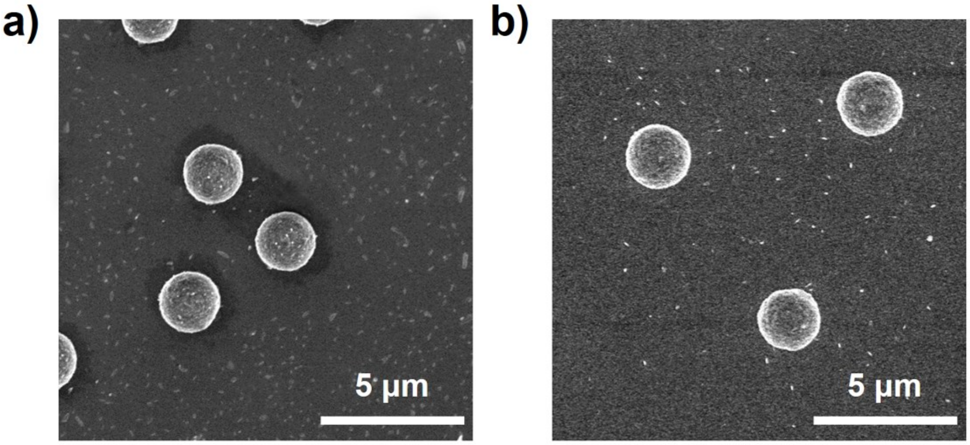

Figure S23. SEM images of **1** (a) and **2** (b) in 0.1 M acetate buffer at pH 6.07 after electrolysis.

References

[1] Apex3, AXScale, and SAINT, version 2017.3-0, Bruker AXS, Inc., Madison, WI, 2017.

[2] G. M. Sheldrick, *Acta Cryst. Sect. C: Struct. Chem.*, 2015, **71**, 3-8.

[3] G. M. Sheldrick, *Acta Cryst. Sect. A. Found. Adv.*, 2015, **71**, 3-8.

[4] N. Tumanova, N. Tumanov, F. Fischer, F. Morelle, V. Ban, K. Robeyns, Y. Filinchuk, J. Wouters, F. Emmerling and T. Leyssens, *CrystEngComm*, 2018, **20**, 7308-7321.

[5] O. V. Dolomanov, L. J. Bourhis, R. J. Gildea, J. A. K. Howard and H. Puschmann, *J. Appl. Cryst.*, 2009, **42**, 339-341.

[6] A. L. Spek, *Acta Cryst. Sect. C. Struct. Chem.*, 2015, **71**, 9-18.

[7] C. Zhang, F. Wang, R. S. Patil, C. L. Barnes, T. Li and J. L. Atwood, *Chem. Eur. J.*, 2018, **24**, 14335-14340.

[8] I. J. Bruno, J. C. Cole, P. R. Edgingtion, M. Kessler, C. F. Macrae, P. McCabe, J. Pearson and R. Taylor, *Acta Cryst. Sect. B. Struct. Sci.*, 2002, **58**, 389-397.

[9] B. C. Gibb, R. G. Chapman and J. C. Sherman, J. Org. Chem. 1996, **61,** 1505-1509.

[10] K. H. Li, D. H. Olson, J. Y. Lee, W. H. Bi, K. Wu, T. Yuen, Q. Xu and J. Li, *Adv. Funct. Mater.*, 2008, **18**, 2205.

[11] a) I. Brown and D. Altermatt, *Acta Cryst. B*, 1985, **41**, 244-247; b) V. Sidey, *Acta Cryst. B*, 2014, **70**, 608-611.

[12] a) B. Weeraswami, P. Bhushanavathi, U. Viplavaprasad and G. N Rao, *Chem. Spec. Bioavailab*., 2013, **25**, 1-5; b) D. Lu, M. E. Wörmann, X. Zhang, O. Schneewind, A. Gründling and P. S. Freemont, *Proc. Natl. Acad. Sci. U.S.A.*, 2009, **106**, 1584-1589.

[13] D. Bansal, A. Mondal, N. Lakshminarasimhan and R. Gupta, *Dalton Trans.*, 2019, **48**, 7918-7927.

[14] J. Li, A. Jiao, S. Chen, Z. Wu, E. Xu and Z. Jin, *J. Mol. Struct.*, 2018, **1165**, 391-100.

[15] a) L. Zhu, J. Du, S. Zuo and Z. Chen, *Inorg. Chem.*, 2016, **55**, 7135−7140; b) J. Du, Z. Chen, S. Ye, B. J. Wiley and T. J. Meyer, *Angew. Chem. Int. Ed*., 2015, **54**, 2073-2078.
